# When Medications Backfire: A Case Report of Rifaximin-Induced Rhabdomyolysis in a Kidney Transplant Patient

**DOI:** 10.7759/cureus.62641

**Published:** 2024-06-18

**Authors:** Munsef Barakat, Salem Vilayet, Genta Uehara, Abubakr Adala, Ahmed I Kamal, Karim Soliman

**Affiliations:** 1 Nephrology, Medical University of South Carolina, Charleston, USA; 2 Transplant Nephrology, Medical University of South Carolina, Charleston, USA; 3 Medicine, Leicester Royal Infirmary, Leicester, GBR

**Keywords:** rifaximin reaction, drug-related side effects and adverse reactions, drug drug interactions, renal transplant recipient, drug induced rhabdomyolysis

## Abstract

Rhabdomyolysis involves significant skeletal muscle injury and destruction, which can be triggered by trauma, intense physical activity, heat, prolonged immobility, certain medications, and endocrine disorders. Rhabdomyolysis in renal transplants can be more complicated, and the prognosis is not well known, especially in the context of coexisting rejection. We present a case of rifampicin-induced rhabdomyolysis with superimposed acute cellular rejection in a kidney transplant patient.

## Introduction

Rhabdomyolysis is a clinical condition characterized by extensive skeletal muscle injury and destruction [1]. It leads to the leakage and release of muscle proteins and enzymes such as myoglobin and creatine phosphokinase (CPK) into the circulation [1]. The first description of the pathological process was reported in the victims of the London Blitz by Bywaters and Beall during World War II [2]. Rhabdomyolysis can be induced by a variety of factors, including trauma and strenuous physical activity, heat, extended immobility, endocrine disorders, autoimmune myositis, toxins, and drugs [3]. Clinical signs typically include muscle soreness, weakness, fever, nausea, vomiting, and tea-colored, dark urine with myoglobinuria [4].

A suggestive history and laboratory results, including elevated levels of lactate dehydrogenase, CPK, myoglobin, and hyperphosphatemia, along with an acid-base imbalance in the presence of acute kidney injury (AKI), comprise the diagnosis of rhabdomyolysis [1].

To our knowledge, rifaximin as a cause of rhabdomyolysis superimposed acute cellular rejection in kidney transplant patients has not been reported in the literature.

Part of this article and the case presentation were previously presented at the American Society of Nephrology meeting on November 4, 2023.

## Case presentation

A 56-year-old male with a complex medical background, including simultaneous pancreas-kidney transplantation, hypertension, diabetes mellitus, seizure disorder, and cholestatic/drug-induced cirrhosis, presented to the ED exhibiting symptoms of progressive generalized weakness and bilateral leg pain localized to the calves. Approximately one month preceding this presentation, the patient had been hospitalized due to hepatic encephalopathy, necessitating the initiation of lactulose and rifaximin therapy, which were subsequently maintained upon discharge to mitigate the risk of recurrence.

The patient reported progressive bilateral leg pain since his previous hospitalization, which had intensified over the preceding two weeks. Describing the sensation as tingling and rated at 9/10 in severity, the pain was localized to his bilateral calves, with greater prominence on the left side. Denying any concomitant symptoms such as fever, chills, nausea, vomiting, diarrhea, chest pain, shortness of breath, abdominal pain, back pain, or urinary symptoms, the patient appeared alert and oriented, presenting no signs of acute distress. Hemodynamically stable, with a blood pressure of 160/80 mmHg, a heart rate of 79 beats per minute, a body temperature of 37.2° C, and a respiratory rate of 18 breaths per minute, the patient exhibited 100% oxygen saturation in room air. Physical examination revealed unremarkable cardiopulmonary and abdominal findings, with no detectable ascites. Notably, bilateral proximal lower limb weakness graded at 2/5 and tenderness in the calves upon palpation were observed, although no lower extremity edema or skin changes were noted. Upper extremity examination was normal, and cranial nerves remained intact, with a negative Babinski sign bilaterally.

Laboratory findings were significant for an elevated serum creatinine level (baseline 1.2-1.5 mg/dl a month prior, 2.4 mg/dL on presentation, and peaking at 4.6 mg/dL). The true trough tacrolimus level was 8.9 ng/mL, with an aldolase level of 35.6 U/L (normal <7 U/L) and a substantially elevated CPK of 14,614 U/L (normal 30-260 U/L) (Table 1). Urinalysis showed gross blood with no blood cells on microscopy (Table 2).

**Table 1:** Full laboratory workup on admission Anti-Jo-1 antibody: anti-histidyl-transfer RNA synthetase; anti-PL-7: anti-threonyl-tRNA synthetase; anti-PL-12 antibody: anti-alanyl tRNA synthetase; anti-EJ antibody: anti-glycyl tRNA synthetase; anti-OJ antibody: anti-isoleucyl tRNA synthetase; anti-SRP antibody: anti-signal recognition particle; anti-MI-2 antibody: one of the dermatomyositis specific antibodies1; anti-U3 RNP: anti-fibrillarin antibody; anti-MDA-5 antibody: anti-melanoma differentiation-associated protein 5 antibody; anti-NXP-2: anti-nuclear matrix protein 2 antibody; anti-TIF-1 gamma antibody: anti-transcriptional intermediary factor 1 gamma antibody; anti-PM/Scl-100 antibody: anti-polymyositis/scleroderma; anti-U-RNP antibody: anti-ribonucleoprotein antibody; anti-SS-A 52kD: anti-Sjögren syndrome A 52-kilodalton antibody; anti-SAE 1 antibody: anti-small ubiquitin-like modifier 1 activating enzyme antibody; anti-U2 RNP antibody: anti-U2 ribonucleoprotein antibody

Parameters	Value	Reference range
Hemoglobin	7.9 g/dl	13-15 g/dl
White blood cell counts	11.7 k/cumm	4-11 k/cumm
Platelets	162 k/cumm	150-400 k/cumm
Serum creatinine	2.4 mg/dl	0.7-1.2 mg/dl
Blood urea nitrogen	27 mg/dl	8-26 mg/dl
Serum sodium	137 mmol/l	135-145 mmol/l
Serum potassium	4.1 mmol/l	3.5-5.1 mmol/l
Serum calcium	8.5 mg/dl	8.4-10.3 mg/dl
Serum bicarbonate	18 mmol/l	22-29 mmol/l
Serum chloride	110 mmol/l	98-108 mmol/l
Creatine phosphokinase	14,614 U/L	30-260 U/L
Ammonia	40 umol/l	18-72 umol/l
Magnesium	2.2 mg/dl	1.6-2.6 mg/dl
Phosphorus	2.3 mg/dl	2.3-4.7 mg/dl
Alanine transaminase	290 U/L	<34 U/L
Aspartate transaminase	924 U/L	<34 U/L
Alkaline phosphatase	424 U/L	35-150 U/L
Total bilirubin	1.7 mg/dl	0.2-1.2 mg/dl
Direct bilirubin	1.2 mg/dl	<0.5 mg/dl
International normalized ratio	1	0.9-1.2
Albumin	1.9 g/dl	3.5-5 g/dl
Anti-Jo-1 antibody	<20 units	<20 units
Anti-PL-7 antibody	Negative	Negative
Anti-PL-12 antibody	Negative	Negative
Anti-EJ antibody	Negative	Negative
Anti-OJ antibody	Negative	Negative
Anti-SRP antibody	Negative	Negative
Anti-MI-2 antibody	Negative	Negative
Anti-U3 RNP	Negative	Negative
Anti-MDA-5 antibody	<20 units	<20 units
Anti NXP-2	<20 units	<20 units
Anti-TIF-1 gamma antibody	<20 units	<20 units
Anti-PM/Scl-100 antibody	<20 units	<20 units
Anti-U1-RNP antibody	<20 units	<20 units
Anti-SS-A 52kD antibody	<20 units	<20 units
Anti-SAE 1 antibody	<20 units	<20 units
Anti-U2 RNP antibody	<20 units	<20 units
Tacrolimus trough level	8.9 ng/ml	4-10 ng/ml
C-peptide	2.94 ng/ml	0.78-5.19 ng/ml

**Table 2:** Urinalysis on admission

Parameters	Value	Reference range
Urine PH	7.5	5-7
Color	Light orange	-
Blood	Large quantity	Negative
Red blood cells	0/HPF	≤1/HPF
White blood cells	5/HPF	<2/HPF
Leukocyte esterase	Negative	Negative
Specific gravity	1,019	1,003-1,030
Urine culture	Negative	-

Other diagnostic investigations, including an MRI of the lumbar spine, a bilateral lower limb Doppler exam, and a kidney ultrasound, were unremarkable. Given the constellation of findings, the patient was diagnosed with rhabdomyolysis.

**Figure 1:**
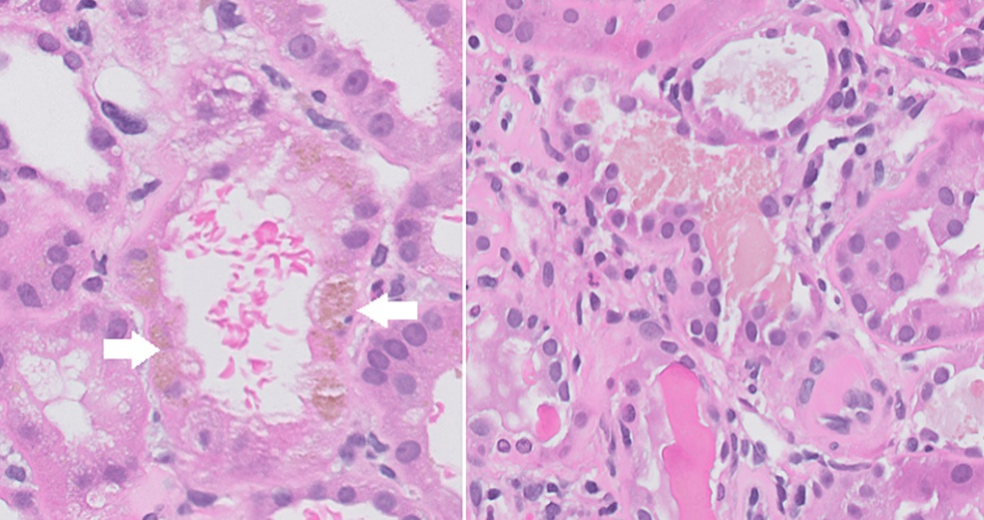
H&E stain of renal tubules notable for the brown pigment in the cytoplasm and granular cast

Management commenced with intravenous crystalloid hydration, aiming to sustain a urine output of approximately 200 mL/hour without necessitating renal replacement therapy. A subsequent transplant kidney biopsy revealed acute tubular injury, pigment nephropathy, and borderline acute cellular rejection, the latter manifested by multifocal mild tubulitis (Figure 1).

The patient was given a pulse dose of intravenous methylprednisolone (500 mg IV per day) for three consecutive days, followed by a rapid tapering prednisone schedule over five days, and continued on tacrolimus and mycophenolate mofetil.

**Figure 2:**
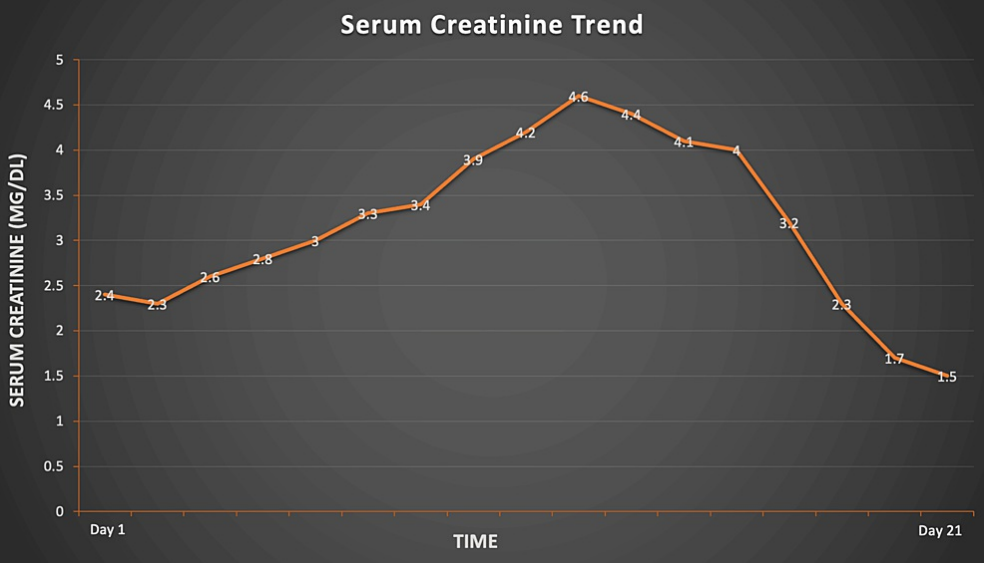
Serum creatinine trends during admission and on follow-up

Extensive workups for myositis and hepatitis panel were unremarkable, suggesting that recent rifaximin use was the most likely cause of rhabdomyolysis. Consequently, rifaximin was discontinued. Over the following two weeks, serum CPK, creatinine, and liver enzymes demonstrated improvement, prompting the patient’s discharge. Subsequent follow-up labs in one week revealed a return to baseline levels of CPK (139 U/L), creatinine (1.5 mg/dL), and liver enzymes, affirming the successful resolution of the episode (Figure 2 shows serum creatinine trends).

## Discussion

Drug-induced muscle injury and rhabdomyolysis have been reported across various medications, statins being one of the most common given their wide use [5]. Statins may lead to a range of skeletal and muscle-related adverse effects, such as myalgia, myositis, and rhabdomyolysis, characterized by a total CPK elevation exceeding 10 times the upper limit of normal, with or without AKI [1]. While rhabdomyolysis induced solely by statins is uncommon, the combination of statins with other medications can lead to notable drug-drug interactions, significantly increasing the risk. This risk is notably elevated with drugs affecting the cytochrome P-450 system, particularly the 3A4 isozyme [5].

The pathophysiology of rhabdomyolysis remains consistent, regardless of the primary cause. It entails dysfunction of the myocyte Na-K ATPase channel and Ca-Na exchanger within the muscle [6]. The initial insult leads to an elevation in intracellular calcium and water, resulting in cellular swelling and dysfunction [6]. The increased intracellular calcium levels trigger the activation of proteases and phospholipases. Alongside local inflammatory activities and increased production of free radicals, these mechanisms contribute to myocyte death and necrosis. This, in turn, releases myoglobin and CPK into the circulation [6,7].

Rifaximin is a broad-spectrum antimicrobial agent used in the prevention and treatment of hepatic encephalopathy [8]. Rifaximin has limited absorption in those with normal gastrointestinal function and is mostly eliminated through stool. However, absorption is improved in individuals with severe cirrhosis [8]. The rifampin component is implicated in the proposed process of rhabdomyolysis induction, whereby it adds to mitochondrial oxidative stress. This increase in oxidative stress increases the likelihood of rhabdomyolysis brought on by statins [9].

In a small, double-phase-2 randomized controlled trial involving 44 patients, the safety of combining simvastatin and rifaximin in individuals with moderate to severe decompensated liver disease (Child-Pugh class B or C) was evaluated. Patients were allocated in a 1:1 ratio to receive either simvastatin 40 mg/day and rifaximin 1,200 mg/day, simvastatin 20 mg/day plus rifaximin 1,200 mg/day, or a placebo for both medications over a period of 12 weeks. Among the high-dose simvastatin group, comprising 16 patients, three (19%) developed rhabdomyolysis and liver injury (two Child-Pugh class C and one Child-Pugh class B). However, the safety profile of the lower simvastatin dose combined with rifaximin was comparable to that of the placebo [10].

While most of the published literature concerning rifaximin-induced rhabdomyolysis involves concurrent use of statins, one case was documented in a patient with alcoholic liver cirrhosis and Child-Pugh Score class C [11]. This patient developed rhabdomyolysis one month after initiating rifaximin 550 mg without concurrent statin use. Interestingly, the patient was previously on atorvastatin, which had been discontinued upon initiating rifaximin [11].

Despite being on atorvastatin for an extended period, our patient never exhibited clinical or subclinical side effects associated with statins. We hypothesize that initiating rifaximin had a synergistic effect on a statin in this patient, resulting in statin-induced myopathy and subsequent rhabdomyolysis following the initiation of rifaximin. Furthermore, a kidney biopsy was essential due to the transplant history and creatinine trend to identify the underlying rejection as an additional cause of the AKI.

Because of their proven survival benefits, statins are frequently prescribed medications for both primary and secondary prevention of dyslipidemia. Like any other medication, statins have both common and less common side effects. This complexity is magnified in patients with polypharmacy and significant comorbidities, which can substantially affect pharmacokinetics [12]. Therefore, initiating new treatments in patients with multiple comorbidities and polypharmacy demands careful attention and reassessment of the indications for long-term prescribed medications.

## Conclusions

Rifaximin is a rare cause of rhabdomyolysis but poses an increased risk of rhabdomyolysis and AKI in patients with advanced liver disease, especially with concomitant use of statins. While clinical suspicion and the diagnosis of rhabdomyolysis as a cause of AKI have been established in our case, it is critical for providers to evaluate and rule out other potential concomitant causes of AKI, particularly in kidney transplant recipients, such as rejection.

Finally, when prescribing new medications to patients with comorbidities, it is essential to weigh the long-term benefits and possible short-term risks. This emphasizes the significance of periodically revisiting the rationale for maintaining chronically prescribed medications.
